# Properties of a Steel Slag–Permeable Asphalt Mixture and the Reaction of the Steel Slag–Asphalt Interface

**DOI:** 10.3390/ma12213603

**Published:** 2019-11-02

**Authors:** Wenhuan Liu, Hui Li, Huimei Zhu, Pinjing Xu

**Affiliations:** 1 College of Materials Science and Engineering, Xi’an University of Architecture and Technology, Xi’an 710055, China; zhuhuimeitj@163.com (H.Z.); xpj6100@163.com (P.X.); 2 Shaanxi Key Laboratory of Architectural Science and Technology, Xi’an 710055, China

**Keywords:** FTIR, asphalt, steel slag, chemical reaction, asphalt–steel slag interface

## Abstract

Steel slag is an industrial solid waste with the largest output in the world. It has the characteristics of wear resistance, good particle shape, large porosity, etc. At the same time, it has good adhesion characteristics with asphalt. If steel slag is used in asphalt pavement, it not only solves the problem of insufficient quality aggregates in asphalt concrete, but can also give full play to the high hardness and high wear resistance of steel slag to improve the performance of asphalt pavement. In this study, a steel slag aggregate was mixed with road petroleum asphalt to prepare a permeable steel slag–asphalt mixture, which was then compared with the permeable limestone–asphalt mixture. According to the Technical Regulations for Permeable Asphalt Pavement (CJJT 190-2012), the permeability, water stability, and Marshall stability of the prepared asphalt mixtures were tested and analyzed. In addition, the high-temperature stability and expansibility were analyzed according to the Experimental Regulations for Highway Engineering Asphalt and Asphalt Mixture (JTG E20-2011). The chemical composition of the steel slag was tested and analyzed by X-ray fluorescence spectrometer (XRF). The mineral composition of the steel slag was tested and analyzed by X-ray diffractometer (XRD). The asphalt was analyzed by Fourier transform infrared spectroscopy (FTIR). The results show that the steel slag asphalt permeable mixture had good permeability, water stability, and Marshall stability, as well as good high-temperature stability and a low expansion rate. The main mineral composition was ferroferric oxide, the RO phase (RO phase is a broad solid solution formed by melting FeO, MgO, and other divalent metal oxides such as MnO), dicalcium silicate, and tricalcium silicate. In the main chemical composition of steel slag, there was no chemical reaction between aluminum oxide, calcium oxide, silicon dioxide, and asphalt, while ferric oxide chemically reacted with asphalt and formed new organosilicon compounds. The main mineral composition of the steel slag (i.e., triiron tetroxide, dicalcium silicate, and tricalcium silicate) reacted chemically with the asphalt and produced new substances. There was no chemical reaction between the RO phase and asphalt.

## 1. Introduction

With the development of road traffic construction in China, the total length of highways in China now exceeds 4.5 million kilometers, of which the total length of expressways is approximately 125,000 kilometers. Asphalt pavement has been widely used in expressways. Approximately 90% of the completed asphalt pavement is designed according to the principle of dense gradation, which has the characteristics of high strength, low-temperature crack resistance, and strong durability [1]. At present, the asphalt concrete used in asphalt pavement uses basalt, limestone, and other natural stones as aggregates, and its usage accounts for approximately 95% of the total amount of asphalt concrete. Due to the large-scale consumption of high-quality aggregates dominated by natural stones in China, natural stone resources are in a state of depletion [2,3]. Determining how to find a material that can replace natural stone as an asphalt concrete aggregate from the perspectives of superior performance, economy, and environmental protection has become an important topic in the field of building materials.

Steel slag is an industrial solid waste type with one of the largest outputs in the world. China has accumulated more than 1 billion tons of steel slag, and its utilization rate is only approximately 20%. Steel slag has the characteristics of abrasion resistance, congruent particle shape, and large porosity, and also has superior adhesion characteristics to asphalt [4,5,6,7,8,9]. Studies on the application of steel slag to asphalt pavement by relevant researchers not only solves the problem of insufficient high-quality aggregates in asphalt concrete but can also fully consider the high hardness and high wear resistance of the steel slag to improve the performance of the asphalt pavement [10,11,12,13,14,15]. Noureldin [16] studied a steel slag asphalt mixture. The results show that the dense gradation asphalt mixture prepared using steel slag as a coarse aggregate and natural sand as a fine aggregate has higher tensile strength and stability. Moreover, the volume expansion rate of the freeze–thaw cycle mixture was less than 1%, which meets the requirements of the “Asphalt Pavement Construction and Acceptance Code”. This British laboratory studied the application of steel slag as an aggregate in asphalt mixtures. The results show that steel slag has a rough surface and wear resistance. The prepared wear-resistant asphalt mixture has a higher friction resistance than basalt asphalt mixtures with the same gradation. Airey [17] studied the performance of a steel slag asphalt mixture. The results show that the surface capsule structure of steel slag assists the asphalt mixture in providing strong road performance; moreover, the dynamic stability, aging resistance, and stiffness of the mixture will be strengthened. Chen [18] studied the application of steel slag as an aggregate in asphalt pavement, showing that steel slag asphalt pavement has strong skid resistance due to the fact of its surface roughness and is superior to natural high-quality aggregate asphalt pavement with the same gradation. These research results generally paid attention to the performance of steel slag and asphalt mixed for pavement engineering, although the chemical composition and mineral composition of steel slag are extremely complex. There has been little research on the interfacial chemical reaction between steel slag and asphalt, especially on the chemical composition, mineral composition, and asphalt chemical reaction of the steel slag.

In this study, the main chemical composition and the main mineral composition of steel slag are taken as research objects. The asphalt–steel slag mixture, asphalt–steel slag chemical composition mixture, and asphalt–steel slag mineral composition mixture were prepared by mixing steel slag raw material, the main chemical composition of steel slag (i.e., alumina, calcium oxide, ferric oxide, and silica), and the main mineral composition of steel slag (i.e., ferroferric oxide, dicalcium silicate, calcium silicate, and the RO phases) with asphalt. The chemical composition of the steel slag was tested with an X-ray fluorescence spectrometer (XRF), the mineral composition of the steel slag was tested by X-ray diffraction (XRD), the structural composition was tested by Fourier transform infrared spectroscopy (FTIR), and the microscopic morphology was tested with a scanning electron microscope (SEM) [19,20,21,22,23]. From the perspective of the main chemical and mineral compositions in the steel slag, the interfacial chemical reaction between the steel slag and asphalt was revealed.

## 2. Experiment

### 2.1. Materials and Reagents

The materials and reagents used in this study were as follows: converter steel slag powder (Shaanxi Longmen Iron and Steel Co., ltd., Xi’an, China), A-70 road petroleum asphalt (Hebei Xingtai Hansheng Asphalt Sales Co., Ltd., Xingtai, China), alumina, calcium oxide, ferric oxide, silica (Tianjin Shengsixin Chemical Co., Ltd., Tianjin, China), ferroferric oxide, dicalcium silicate, calcium silicate, RO phase (homemade), and deionized water as the experimental water.

### 2.2. Experimental Method

The preparation of the steel slag asphalt permeable mixture was as follows: Firstly, 1202.29 g of steel slag particle aggregate with a grading specification of 4.75–9.5 mm and 105 g of steel slag micro powder filler was weighed, mixed, dried to a constant weight in an oven at 170 °C, and poured into an E10-H asphalt mixer. Then, 4.19 g of the high modulus agent was added and uniformly mixed for 180 s. Then, 87.91 g of the A-70 road petroleum asphalt was added and uniformly mixed for 180 s. 

The preparation of the asphalt–steel slag chemical composition mixture was as follows: Road petroleum asphalt and steel slag chemical compositions (i.e., alumina, calcium oxide, ferric oxide and silica) were weighed and prepared via an electronic analysis balance according to a mass ratio of 4:1. After the road petroleum asphalt was heated to a flowing state, alumina, calcium oxide, ferric oxide, and silica were added to the asphalt in a flowing state. The asphalt and each chemical component were evenly stirred at 175 °C to 190 °C for 60 min by using an asphalt stirrer to obtain the asphalt–alumina mixture, asphalt–calcium oxide mixture, asphalt–ferric oxide mixture, and asphalt–silica mixture.

The preparation of the asphalt–steel slag mineral composition mixture was as follows: Road petroleum asphalt and the steel slag mineral composition (i.e., ferroferric oxide, dicalcium silicate, calcium silicate, and RO phases) were weighed and prepared by an electronic analysis balance according to a mass ratio of 4:1. After the road petroleum asphalt was heated to a flowing state, ferroferric oxide, dicalcium silicate, calcium silicate, and RO phases were added to the asphalt in a flowing state. The asphalt and each mineral component were evenly stirred at 175 °C to 190 °C for 60 min using an asphalt stirrer to obtain the asphalt–ferroferric oxide mixture, asphalt–dicalcium silicate mixture, asphalt–calcium silicate mixture, and asphalt–RO phase mixture.

The pure RO phase was a −8 μm powder with a purity of ≥99.8% extracted from steel slag powder. We removed all the strong magnetic minerals (Fe and Fe_3_O_4_) from the finely ground steel slag powder by repeated magnetic separation with a low magnetic field (63 mT); then, the EDTA–DEA–TEA (Disodium ethylenediamine tetraacetate-diethylamine-triethanolamine) solution was used to dissolve and remove all calcium-containing minerals. Finally, a high-intensity magnetic field (1 T) was used for repeated magnetic separation to remove the refractory impurities in the dissolved residue to obtain a pure RO phase sample.

### 2.3. Performance Test and Characterization

The permeability, water stability, and Marshall stability of the prepared asphalt mixtures were tested according to the Technical Specifications for Permeable Asphalt Pavement (CJJT 190-2012 [24]), and the high-temperature stability and expansibility were tested according to the Experimental Specifications for Highway Engineering Asphalt and Asphalt Mixtures (JTG E20-2011 [25]).

The chemical composition was tested by an S4 PIONEER X-ray fluorescence analyzer (Bruker Corporation, Karlsruhe, Baden-Württemberg, Germany), the mineral composition was tested by a D-MAX/2500 X-ray diffractometer (Rigaku Corporation, Zhaodao, Tokyo, Japan), the structural composition was tested by a spectrum two Fourier infrared spectrometer(PERKINELMER Corporation, Waltham, MA, USA), and the microscopic morphology was tested by a Quanta 2000B scanning electron microscope (FEI Corporation, Hillsboro, OR, USA).

## 3. Results and Discussion

### 3.1. Performance Analysis

Table 1 shows the test results for the permeability, water stability, and Marshall stability of the prepared asphalt mixtures. The permeable steel slag–asphalt mixture had a permeability coefficient of 60.61 mL/s, a 0.5 h stability of 9.12 kN, a 48 h stability of 8.27 kN, and a Marshall stability of 9.12 kN, indicating that the permeable steel slag–asphalt mixture had good permeability, water stability, and Marshall stability. 

Table 2 shows the test results of the high temperature stability and expandability. It can be seen that the steel slag–asphalt permeable mixture had a dynamic stability of 6350 and an expansion rate of 0.49%, indicating that the steel slag–asphalt permeable mixture had good high temperature stability and a low expansion rate.

### 3.2. Analysis of the Chemical Composition of the Steel Slag–Asphalt Mixture

Table 3 shows the XRF test results of the steel slag. It can be seen from the table that the main chemical compositions of the steel slag were SiO_2_, Al_2_O_3_, CaO, and Fe_2_O_3_, among which the steel slag contained CaO, resulting in the presence of a certain amount of f-CaO. The reaction between f-CaO and H_2_O can generate hydroxide, which will promote the volume expansion of the steel slag asphalt permeable mixture. If the stress during expansion is too large, the steel slag will crack, and the mechanical properties of the steel slag–asphalt permeable mixture will be affected. Table 4 shows the steel slag mechanical properties that meet the requirements of a high-quality aggregate for roads.

**Figure 1:**
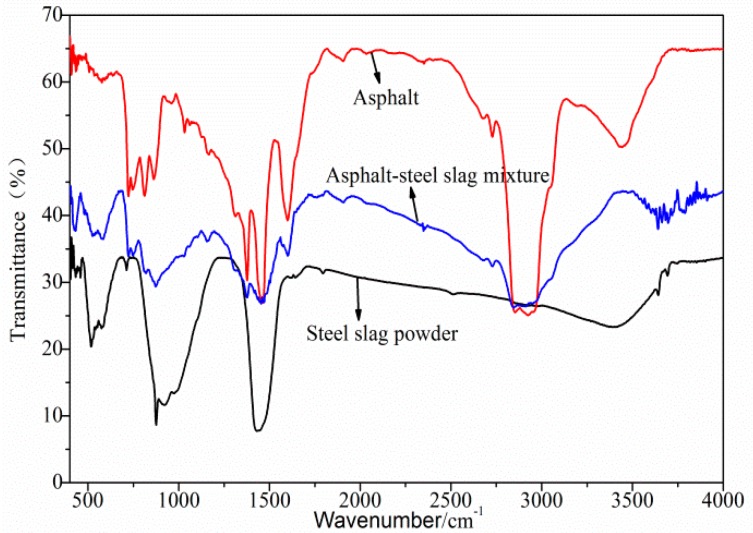
FTIR test results of the asphalt–steel slag mixture.

Figure 1 and Table 5 show the FTIR test results of the asphalt–steel slag mixture. Road petroleum asphalt has a wide and weak absorption peak caused by N–H or O–H bonds between 3200 cm^−1^ and 3700 cm^−1^, which is due to the interactions among the component molecules. There was a wide and scattered absorption peak at 2500~3200 cm^−1^, which is characteristic of typical carboxylic acid. The strong absorption peaks at 2920 cm^−1^ and 2850 cm^−1^ are the C–H stretching vibrations of alkanes and cycloalkanes. The absorption peak at 2950 cm^−1^ was a methyl-CH_3_ telescopic vibration, and methylene -CH_2_– had the strongest absorptions at 2920 cm^−1^ and 2850 cm^−1^. The absorption band between 1560 cm^−1^ and 1640 cm^−1^ was an N–H deformation vibration, which proves the existence of amine compounds. The two strong absorption peaks at 1460 cm^−1^ and 1375 cm^−1^ were caused by the stretching vibration of the asymmetric bond of C–CH_3_ and the symmetric bond of –CH_2_–. The absorption peaks from 1400 cm^−1^ to 1420 cm^−1^ were caused by a C–N stretching vibration. These are characteristic absorption peaks of amides, indicating the existence of amide functional groups. The absorption band between 1030 cm^−1^ and 1280 cm^−1^ was caused by the expansion and contraction vibration of aliphatic amine C–N; thus, it contained aliphatic amine functional groups. At 1280 cm^−1^, there was an Ar–O stretching vibration absorption peak, indicating the existence of ether compounds. The strong absorption peak at 1030 cm^−1^ belonged to the vibration of the S=O functional group of sulfoxides. The absorption band at 650~900 cm^−1^ was caused by a bending vibration absorption outside the N–H moiety. The values of 800 cm^−1^ and 860 cm^−1^ represent the aromatic external absorption frequency and, thus, contained aromatics. The broad and weak absorption peaks of the converter steel slag powder caused by the N–H or O–H stretching vibrations between 3200 cm^−1^ and 3700 cm^−1^ were due to the interactions among the component molecules. The absorption band between 1300 cm^−1^ and 1600 cm^−1^ was caused by the –CH_3_ symmetric deformation vibration and ring expansion vibration, indicating that aromatic compounds were contained within. The bending vibration outside this moiety, the stretching vibration of Si–O, and the bending vibration of Si–H at 750 cm^−1^ to 1200 cm^−1^ were C–H, indicating the existence of silicate compounds. The absorption band between 750 cm^−1^ and 500 cm^−1^ was caused by the C–H moiety external bending vibration, N–O bending vibration, and C–S stretching vibration. The band was caused by the S–S stretching vibration at 400 cm^−1^~500 cm^−1^, indicating a sulfate compound. The absorption peak at 3407 cm^−1^ was caused by the stretching vibration of hydrogen, and the absorption peak at 1430 cm^−1^ was the bending vibration of –CH_2_. The absorption peak at 921 cm^−1^ was caused by the –CH(CH_3_)_2_ bending vibration of alkane. The absorption peak at 710 cm^−1^ was the C–H moiety external bending vibration of meta-disubstituted aromatic hydrocarbons. The absorption peak near 500 cm^−1^ was caused by the deformation vibration of C–N–O.

Furthermore, Figure 1 shows that, on the one hand, after the interaction between the road petroleum asphalt in the asphalt–steel slag mixture and the converter steel slag powder, most of the absorption peaks in the asphalt–steel slag mixture were the superposition of the absorption peaks of the road petroleum asphalt and the converter steel slag powder. No obvious new stretching vibration peaks or deformation vibration peaks were generated, proving that physical action was the main action between the road petroleum asphalt and the converter steel slag powder. On the other hand, the broad and weak absorption peak of the asphalt–steel slag mucilage at 3200 cm^−1^ to 3700 cm^−1^ were due to the disappearance N–H or O–H bonds, and the absorption peaks at 2500 cm^−1^ to 3200 cm^−1^ decreased, which indicates that the intermolecular interaction of road petroleum asphalt components was weakened. There was a new absorption peak between 3500 cm^−1^ and 3750 cm^−1^ which was caused by the N–H stretching vibration of amines and amides and SiO–H stretching vibrations. This shows that the interaction between the road petroleum asphalt and the converter steel slag powder was also caused by a certain chemical reaction.

**Figure 2:**
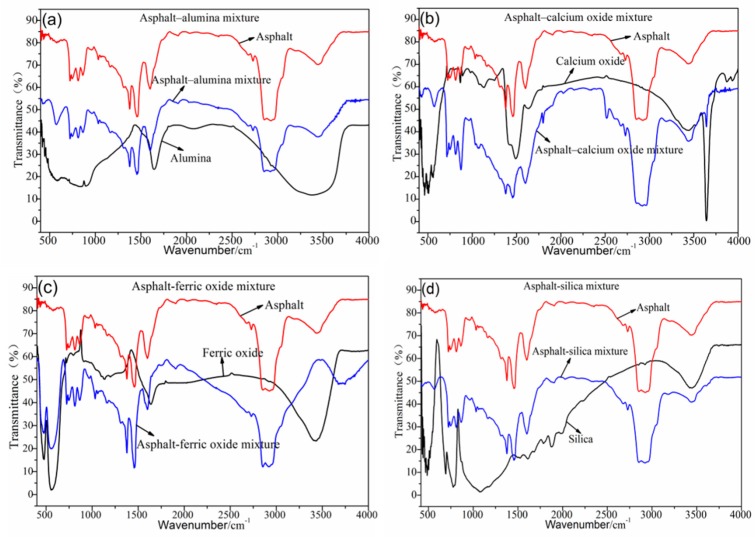
FTIR results of the chemical composition of the asphalt–steel slag mixture. (**a**) FTIR results of Asphalt-alumina mixture, (**b**) FTIR results of Asphalt-calcium oxide mixture, (**c**) FTIR results of Asphalt-ferric oxide mixture, (**d**) FTIR results of Asphalt-silica mixture.

Figure 2a–d shows the FTIR test results of the asphalt–steel slag chemical composition mixture. Most absorption peaks in the asphalt–silica mixture, asphalt–alumina mixture, and asphalt–calcium oxide mixture were the superposition of the absorption peaks of road petroleum asphalt and the absorption peaks of the steel slag’s chemical composition (alumina, calcium oxide, and silica). This dynamic indicates that no chemical reaction occurs between alumina, calcium oxide and silica, and road petroleum asphalt. The asphalt–ferric oxide mixture has a new absorption peak at 3645 cm^−1^, namely, the vibration peak generated by the SiO–H stretching vibration, which indicates that ferric oxide and road petroleum asphalt have a chemical reaction and generate a new organosilicon compound.

### 3.3. Analysis of the Mineral Composition of the Steel Slag–Asphalt Mixture

**Figure 3:**
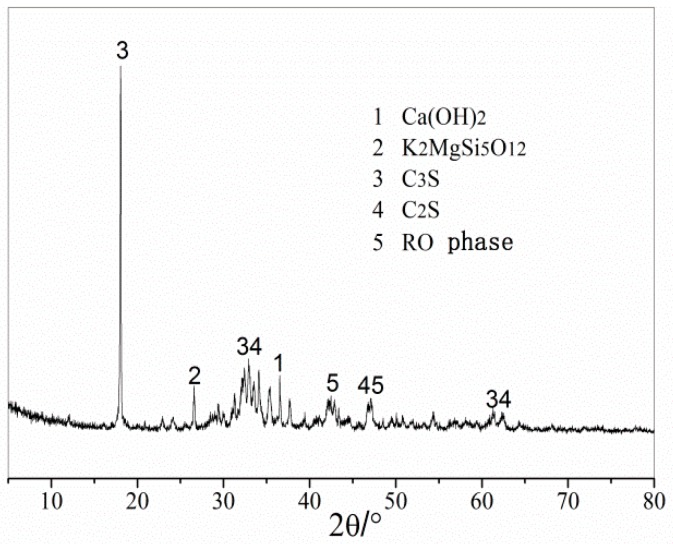
XRD test results of steel slag aggregate.

Figure 3 shows the XRD results of the steel slag aggregate. The main mineral components of the steel slag aggregate were calcium hydroxide, the RO phase, dicalcium silicate, and tricalcium silicate. The steel slag aggregate contained a certain amount of calcium hydroxide, which endowed the steel aggregate powder with strong alkalinity and could react with the anhydride in asphalt to enhance the bonding force between the steel slag aggregate and asphalt.

**Figure 4:**
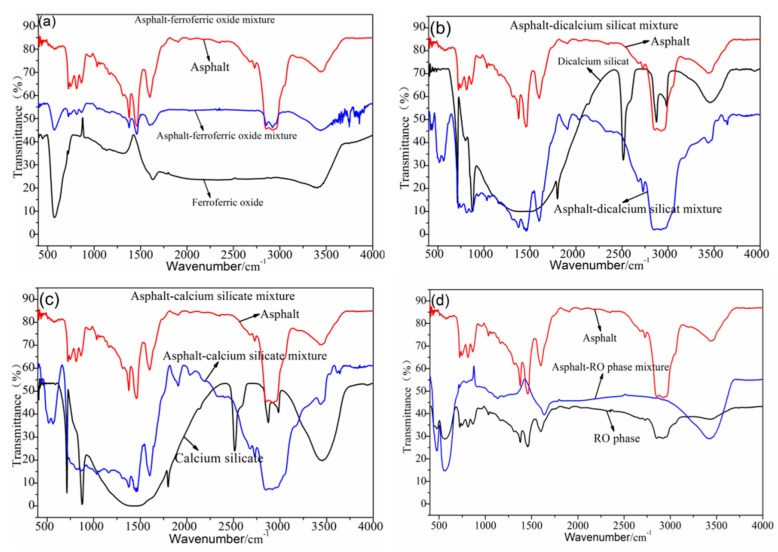
FTIR test results of the steel slag mineral composition–asphalt mixture. (**a**) FTIR test results of Asphalt-ferroferric oxide mixture, (**b**) FTIR test results of Asphalt-dicalcium silicate mixture, (**c**) FTIR test results of Asphalt-calcium silicate mixture, (**d**) FTIR test results of Asphalt-RO phase mixture.

Figure 4 shows the FTIR test results of the asphalt–steel slag mineral composition mixture. The asphalt–ferroferric oxide mixture had relatively weak new absorption peaks at 3500 cm^−1^~3750 cm^−1^, which were generated by the N–H stretching vibration of amines and amides and the SiO–H stretching vibration. This dynamic indicates that the ferroferric oxide had a chemical reaction with the road petroleum asphalt and generated a new organosilicon compound. Most absorption peaks in the asphalt–dicalcium silicate mixture and asphalt–calcium silicate mixture were the superposition of the absorption peaks of the road petroleum asphalt and the absorption peaks of the steel slag mineral composition (dicalcium silicate and calcium silicate); no obvious new stretching vibration peaks or deformation vibration peaks were generated. A new absorption peak appeared at 3645 cm^−1^, which was caused by the SiO–H stretching vibration, indicating that dicalcium silicate and calcium silicate have chemical reactions with the road petroleum asphalt and generate new substances. Most absorption peaks in the asphalt–RO phase mixture were the superpositions of the absorption peaks of the road petroleum asphalt and the RO phase, indicating that there was no chemical reaction between the road petroleum asphalt and RO phase.

### 3.4. Analysis of the Reaction of the Steel Slag–Asphalt Interface

**Figure 5:**
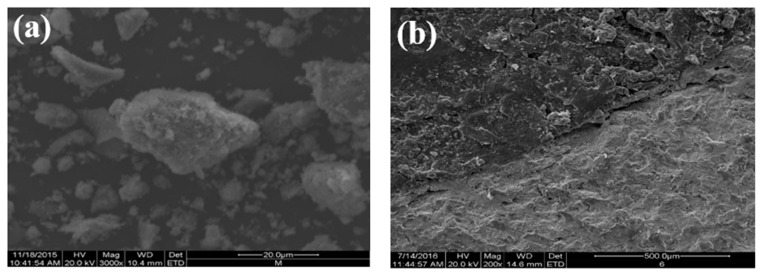

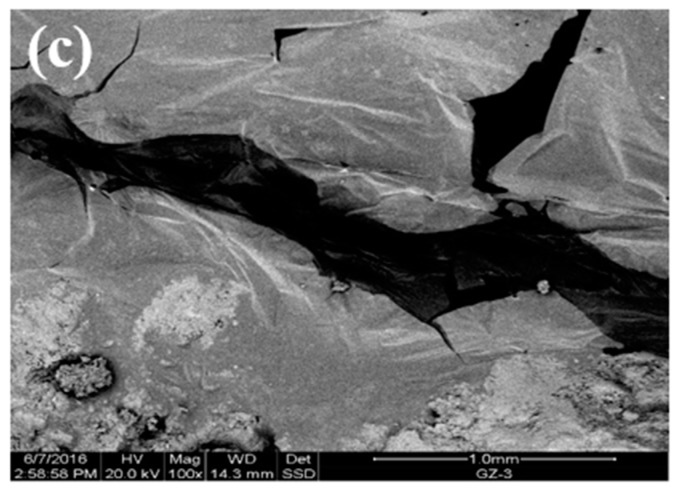
Morphologies: (**a**) SEM of the steel–slag aggregate; (**b**) SEM of the asphalt–steel slag mineral composition mixture; (**c**) SEM of the steel slag–asphalt interface.

Figure 5 shows the SEM results. The steel slag aggregate had a rough surface and pore structure (Figure 5a). There were pits and grooves on the surface of the asphalt–steel slag mineral composition mixture (Figure 5b) because the pore structure of the steel slag was conducive to the formation of a certain embedding depth between the asphalt and the steel slag aggregate and thereby improved the interface adhesion. In Figure 5c, Asphalt in the steel slag–asphalt interface permeated through pores on the surface of the steel slag aggregate to form a steel slag–asphalt composite phase. This formation process involved the physical wrapping of the steel slag by the asphalt, as well as chemical reactions between the asphalt and the steel slag aggregate, indicating that the steel slag aggregate with a rough surface and pore-like structure could form a skeleton-like system with asphalt, increase interface strength, and improve water permeability and the high-temperature stability of the steel slag asphalt permeable mixture.

## 4. Conclusions

On the basis of the test results and the performance survey conducted in this study, the following conclusions can be drawn. The chemical and mineral compositions of steel slag are extremely complex. The main chemical compositions are alumina, calcium oxide, ferric oxide, and silica. The main mineral compositions are ferroferric oxide, the RO phase, dicalcium silicate, and calcium silicate. There is no chemical reaction between alumina, calcium oxide, silica, and road asphalt in the main chemical composition of the steel slag. Iron oxide and road asphalt undergo a chemical reaction and generate a new organosilicon compound. Ferroferric oxide, dicalcium silicate, and calcium silicate in the main mineral composition of the steel slag have chemical reactions with the road petroleum asphalt and generate new substances. No chemical reaction occurs between the RO phase and the road petroleum asphalt.

## Figures and Tables

**Table 1:** Test results of the permeability, water stability, and Marshall stability.

Water Permeability			Water Stability			Marshall Stability		
Volume /mL	Time /s	coefficient of permeability /(mL/s)	Stability of 0.5h /kN	48 h stability /kN	Residual degree of immersion /%	Marshall stability /kN	Flow value /mm	Void ratio /%
400	6.6	60.61	9.12	8.27	90.68	9.12	23.3	19.64

**Table 2:** Test results of the high temperature stability and expandability.

High Temperature Stability	Expansibility		
Dynamic stability (60 °C, 1 h, Times /mm)	Initial volume /mm^3^	Final volume /mm^3^	Expansion rate /%
6350	509235	511721	0.49

**Table 3:** XRF test results of steel slag powder (*wt*%).

CaO	Fe_2_O_3_	SiO_2_	Al_2_O_3_	MgO	MnO	P_2_O_5_	TiO_2_	SO_3_	Na_2_O	K_2_O	Others
44.83	21.65	14.38	5.48	3.42	1.94	0.83	0.57	0.23	0.05	0.04	6.58

**Table 4:** Mechanical properties of steel slag.

Test Item	Measured Value	Technical Indicators	Normative References of the Tests
Apparent relative density (g/cm^3^)	3.39	≥2.90	JTG E20-2011
Water absorption (%)	2.4	≤3.0	JTG E20-2011
Needle particle content (%)	4.56	≤12	JTG E20-2011
Aggregate crushing value (%)	13.9	≤26	JTG E20-2011
Water washing method <0.075 mm (%)	0.2	≤1.0	JTG E20-2011
Los Angeles abrasion loss (%)	13.2	≤26	JTG E20-2011
Incorruptibility (%)	2.6	≤12	JTG E20-2011
Soaking expansion rate (%)	1.2	≤2.0	JTG E20-2011
Adhesion to Asphalt (%)	5	≥4	JTG E20-2011
f*-*CaO (%)	1.7	≤3.0	JTG E20-2011

**Table 5:** FTIR peak wavenumbers of the asphalt–steel slag mixture.

Sample	FTIR Peak Wavenumbers (cm^−1^)									
Asphalt	3200 and 3700	2500~3200	2920 and 2850	2950	1560 and 1640	1460 and 1375	1400~1420	1030 and 1280	650~900	800 and 860
Steel slag powder	3200 and 3700	1300 and 1600	750~1200	750 and 500	400~500	3407	1430	921	710	500
